# Development and Validation of Pretreatment Serum Total Bilirubin as a Biomarker to Predict the Clinical Outcomes in Primary Central Nervous System Lymphoma: A Multicenter Cohort Study

**DOI:** 10.3390/cancers15184584

**Published:** 2023-09-15

**Authors:** Jiazhen Cao, Shengjie Li, Danhui Li, Wei Hua, Lin Guo, Zuguang Xia

**Affiliations:** 1 Department of Clinical Laboratory, Fudan University Shanghai Cancer Center, Shanghai 200032, China; 21211230005@m.fudan.edu.cn; 2 Department of Oncology, Shanghai Medical College, Fudan University, Shanghai 200032, China; 3 Department of Neurosurgery, Huashan Hospital, Fudan University, Shanghai 200040, China; lishengjie6363020@163.com (S.L.); hs_huawei@126.com (W.H.); 4 Institute of Neurosurgery, Fudan University, Shanghai 200040, China; 5 Shanghai Key Laboratory of Brain Function Restoration and Neural Regeneration, Shanghai 200040, China; 6 Department of Clinical Laboratory, Eye & ENT Hospital, Shanghai Medical College, Fudan University, Shanghai 200031, China; 7 Department of Pathology, RenJi Hospital, School of Medicine, Shanghai Jiaotong University, Shanghai 200127, China; danhuili1002@163.com; 8 Department of lymphoma, Fudan University Shanghai Cancer Center, Fudan University, Shanghai 200032, China

**Keywords:** serum total bilirubin, biomarker, cohort study, primary central nervous system lymphoma, diffuse large B-cell lymphoma, prognosis

## Abstract

**Simple Summary:**

The prognosis of patients with primary central nervous system lymphoma (PCNSL) is poor due to the high recurrence rate and lack of consensus on the optimal treatment for refractory or relapsed diseases. Despite having good prognostic scores, such as an MSKCC score or IELSG score, none of them are able to fully explain the clinical outcomes of patients with PCNSL, and they are complicated at the same time. Thus, there is an urgent need to develop simple and readily available parameters that help to stratify the prognosis of patients, especially those at high risk, and provide them with appropriate treatment measures. For the first time, this study identified that pretreatment serum total bilirubin (STB) could predict the prognosis of patients with PCNSL receiving high-dose methotrexate-based combination immunochemotherapy. The results from this study demonstrate that STB is a simple, cost-effective prognostic biomarker that clinicians can apply easily and quickly into routine practice.

**Abstract:**

Primary central nervous system lymphoma (PCNSL) is a predominantly aggressive neoplasm isolated to the central nervous system or vitreoretinal space. Bilirubin is an important biomarker reflecting hepatic function and oxidative stress status that is associated with the occurrence and development of various tumors. However, its prognostic role in PCNSL has yet to be evaluated. Therefore, we conducted a prospective–retrospective study to analyze the predictive value of serum total bilirubin (STB) in PCNSL patients. The association between the pretreatment STB and clinical outcomes in PCNSL was developed in the discovery cohort (retrospective [n = 44] and prospective [n = 45]) and validated in an independent retrospective cohort (n = 69). A generalized additive model, Kaplan–Meier curve, and Cox analysis were applied. In the discovery cohort, the STB showed a linear relationship with overall survival (OS, *p* = 0.011) and progression-free survival (PFS, *p* = 0.0476). The median STB level of 12.0 µmol/L was determined as the cutoff value to predict the clinical outcomes with area under the receiver operating characteristic curve (AUROC) values of 0.9205 and 0.8464 for OS and PFS, respectively. The median STB level resulted in similar accuracy for predicting the clinical outcomes in the validation cohort with AUROC values of 0.8857 and 0.8589 for OS and PFS, respectively. In both the discovery and validation cohorts, the Kaplan–Meier survival curve and Cox regression analysis showed that the upper median STB groups showed significantly worse OS than the lower median STB groups. In conclusion, the pretreatment STB could be considered a novel biomarker to predict the clinical outcomes in patients with PCNSL receiving high-dose methotrexate-based combination immunochemotherapy.

## 1. Introduction

Primary central nervous system diffuse large B-cell lymphoma is a rare and aggressive form of extranodal non-Hodgkin lymphoma (NHL) with a predilection for the elderly and accounts for >95% of primary central nervous system lymphomas (PCNSLs) [1]. Currently, high-dose methotrexate (HD-MTX)-based chemotherapy is the first-line treatment for patients with PCNSL [2]. Despite the high tumor response rate to initial therapy, approximately one-third of the patients do not respond to first-line therapy. Up to 60% of patients will eventually relapse, and the prognosis is poor [3], and the optimal treatment for refractory or relapsed PCNSL has yet to reach a consensus [4,5]. Currently, the International Extranodal Lymphoma Study Group risk score [6] and Memorial Sloan Kettering Cancer Center nomogram [7] are the most commonly used comprehensive prognostic stratification indices for PCNSL. Despite having good prognostic scores, none of them can fully explain the prognosis of patients with PCNSL, and they are complicated. Thus, it is necessary to determine simple, and readily available parameters to stratify the prognosis of patients diagnosed with PCNSL, especially those at high risk, and to provide appropriate treatment measures.

Serum total bilirubin (STB) is primarily a metabolic product of heme in hemoglobin [8,9,10,11]. Since it was first reported by Stocker et al. [12] and Najib et al. [13], numerous experimental and clinical studies have found that bilirubin exerts potential endogenous antioxidant and anti-inflammatory effects in disease prevention [14,15,16,17,18,19]. Because chronic inflammation plays a significant role in tumor promotion [20], various studies have investigated the association between pre-diagnostic bilirubin levels and the risk of cancer with inconsistent results. Seyed Khoei et al. [21] performed a nested case–control study (n = 1386) and discovered a positive correlation between higher UCB levels and colorectal cancer (CRC) risk in males, whereas an inverse association was observed in females. In another population-based cohort study (n = 5487), no association was found between the baseline serum bilirubin level and CRC incidence [22]. More recently, a large prospective cohort study found that STB was positively associated with the risks of both melanoma and breast cancer [23].

To our knowledge, there is currently no research on the prognostic significance of bilirubin in patients with PCNSL. Therefore, we aimed to determine whether the STB level may play an important prognostic role in the clinical practice of PCNSL and further determined whether the pretreatment level of STB could be considered a biomarker to predict the clinical outcomes in patients newly diagnosed with PCNSL receiving MTX-based combination immunochemotherapy treatment in a multicenter cohort study consisting of discovery and validation cohorts.

## 2. Materials and Methods

### 2.1. Patients

In total, 228 patients with PCNSL were enrolled in this prospective–retrospective study. All of them were from three independent cohorts in Shanghai, including the Shanghai Cancer Center, Huashan Hospital, and Renji Hospital cohorts. This study was approved by the Ethics Committee (certification no.1612167-18 (26 December 2016) and certification no. 2022-529 (06 April 2022)). Informed oral and written consent were obtained from each patient. This study was conducted in accordance with the principles of the Declaration of Helsinki.

Eligible participants in the current study were those who underwent 2-deoxy-2-[18]fluoro-D-glucose (18FDG) PET-CT (positron emission tomography–computed-tomography) as well as bone marrow biopsy and had histological confirmation of PCNSL. Individuals found to be HIV-positive, EBV-positive, or HBV-positive were excluded. Other exclusion criteria were as follows: any tumors; severe impairment of cardiac function, heart failure, or symptomatic coronary artery disease; third-space fluid; prior radiotherapy or chemotherapy; uncontrolled infection; multiple organ failure or immunocompromised status; and pregnancy. Patients without baseline measurements of the STB level were also excluded, resulting in a final study sample of 158 participants.

For the cases included, clinical variables and laboratory data were collected from the patients’ electronic medical records system from all three institutions. We included the following baseline characteristics at the time of diagnosis: age; sex; alanine aminotransferase (ALT), aspartate aminotransferase (AST), gamma-glutamyl transferase (gamma-GT); and lactate dehydrogenase levels; height; weight; hypertension; diabetes mellitus; medical history; Eastern Cooperative Oncology Group performance status; and tumor subsite. Peripheral venous blood samples were collected before the initiation of treatment for each constitution. Prognostic scores, including the IPI score, ECOG performance, MSKCC score, and the IELSG score, were also collected, and relationship between these data and clinical outcomes had been published previously [24,25].

### 2.2. Diagnostic Criteria

In accordance with the 2016 World Health Organization criteria, all enrolled patients underwent histological diagnosis through CT or magnetic resonance imaging (MRI)-guided stereotactic biopsy or neurosurgical resection, and CD20 positivity was required for PCNSL [26]. Patients also underwent contrast-enhanced MRI, which is the recommended imaging modality before treatment, and those with brain parenchymal lesions showing homogeneous contrast enhancement were also eligible. Furthermore, all patients underwent 18FDG PET/CT combined with bone marrow aspiration.

### 2.3. Follow-Up and Treatment

Follow-up investigations were performed at regular intervals during outpatient visits (3-month intervals in 1–2 years and 6-month intervals in years after then until death) and included clinical examination, posttreatment contrast-enhanced MRI, and clinical laboratory measurements, such as hematology, electrolytes, and renal and liver function, which were measured before and after each administration of MTX. If recurrence was suspected, contrast-enhanced MRI was performed immediately. If there was evidence of disease progression, patients were followed up once every 3 months for their survival status. In cases of second remission, patients should return to their previous follow-up schedule. Overall survival (OS) was defined as the time from diagnosis to death from any cause or the last follow-up, and progression-free survival (PFS) was defined as the time from diagnosis to tumor progression or recurrence. The primary endpoint of the study was OS, and the secondary endpoint was PFS.

All patients enrolled received methotrexate-based combination immunochemotherapy (MTX + ibrutinib/zanubrutinib + rituximab (R) with or without dexamethasone (DXMS)). The detailed information about treatment is as follows: On day 1, for those aged ≤ 65 years old, HD-MTX was administrated at a dose of 8 g/m^2^. For those aged >65 years old, MTX dose was reduced to 5 g/m^2^. Patients aged ≥70 years or who were unfit were treated with MTX dose at only 3.5 g/m^2^. On days 1–3, dexamethasone was administrated at dose of 15 mg/d. Proper adjustments might have been made according to patient’s renal function. At least 72 h prior to MTX administration, prehydration and alkalinization were initiated. Urine volume was strictly monitored and maintained at 3000 mL/24 h. A total of 24 h after MTX initiation, standard leucovorin rescue was given at a dose of 15 mg/m^2^ once every 6 h, 8 times in total. The dose of leucovorin or intravenous fluid hydration and alkalinization rate were increased upon occurrence of delayed elimination. Additional chemotherapy cycles were administrated every 3 weeks for at least 8 cycles [27].

Censoring data were defined as the study subject who experienced other events or survival outcomes beyond this study, and the survival time of the endpoint event cannot be clearly observed and recorded at the end of this study.

Several reasons for censoring data are listed below:

(1) By the deadline for this study, the endpoint event had yet to occur, and the study subjects were still alive.
(2) The study subject lost contact due to relocation, change of phone number, and other reasons, resulting in a loss of follow-up. It is not possible to clearly observe whether the study subject had an endpoint event and the specific time of occurrence.
(3) Due to other reasons, including lack of cooperation from the research subjects or changes in treatment plans by doctors, the study subjects withdrew from this study midway and were unable to continue follow-up observation.

### 2.4. Laboratory Measurement of STB

Peripheral intravenous blood samples were obtained from the patients enrolled before treatment initiation. The STB level was measured using a Roche Cobas 8000 c702 automatic biochemical analyzer (Basel, Switzerland). The unit of the STB level was µmol/L. Four to five milliliters of blood samples were obtained in the morning after 8 h of fasting, stored in the dark, and measured within 2 h after blood collection. Internal controls were monitored and analyzed every day for a total of 10 years, and monthly coefficients of variation (CVs) ranged from 0.900% to 2.300%. No significant changes were observed in CVs. The discovery and validation cohorts followed the standard venous blood sampling procedure.

### 2.5. Statistical Analysis

Continuous variables were presented as median (interquartile range [IQR]), and categorical variables were expressed as frequency (percentage). Data normality was assessed using the Kolmogorov–Smirnov test. The independent Student’s *t*-test, Wilcoxon rank-sum test, chi-square test, one-way ANOVA test, and Kruskal–Wallis rank test were used to compare the characteristics of patients between or among different groups according to different types of variables.

Survival curves were constructed using the Kaplan–Meier method and compared using the log-rank test to analyze prognostic significance. Univariate and multivariate Cox proportional hazards regression models were used to evaluate hazard ratios (HRs) and 95% confidence intervals (CIs) for STB, as well as other potential confounders, including age; ALT, AST, and gamma-GT levels; sex; diabetes; body mass index (BMI); and hypertension, which could potentially affect OS or PFS. Correlations between pretreatment STB and the risk of survival outcomes were applied using a generalized additive model (GAM) [28] and locally weighted regression (Loess) nonparametric models. Statistical analyses were performed using R statistical programming environment (v4.1.0; R Core Team 2021, R Foundation for Statistical Computing, Vienna, Austria). In detail, packages mgcv (v1.8-31) [29] were used to perform the GAM to verify a potential nonlinear relationship between pretreatment STB and prognosis. The gamm() function from the mgcv package was used to perform generalized additive mixed modeling to account for the random effect caused by the clustered (nested) structure of the data. Compared to traditional linear models, the combined application of the GAM and Loess was more rigorous for exploring the relationship between the pretreatment STB levels and prognosis.

Receiver operating characteristic (ROC) curves were generated to define the sensitivity, specificity, and area under the ROC (AUROC) using GraphPad Prism 8 software for Windows.

Spearman’s correlation was applied to analyze the relationship between STB and clinical outcomes. Logistic regression analyses were conducted to assess the association between pretreatment STB levels and clinical outcomes. Pearson’s correlation analysis was conducted to analyze the correlation between STB and age. Fine and Grey’s competing risk regression model was used to further explore the relationship between STB and prognosis.

Statistical analyses were performed using SPSS software for Windows version 23.0, and graphs were created using GraphPad Prism 8 software for Windows. A *p*-value of <0.05 indicated statistical significance. All quoted *p*-values were two-sided.

## 3. Results

### 3.1. Patient Characteristics

**Figure 1:**
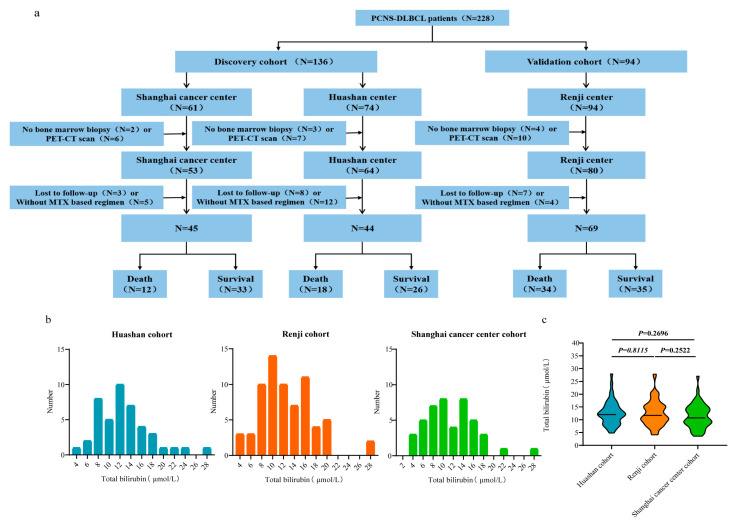
The flowchart of the prospective–retrospective analysis (**a**). Frequency distribution of pretreatment serum total bilirubin (STB) in every constitution (**b**). Violin plots of STB in three constitutions (**c**).

From 1 January 2010 to 31 December 2020, 228 newly diagnosed PCNSL patients from three institutions were enrolled (Figure 1a). The discovery cohort consisted of patients from the Shanghai Cancer Center and Huashan Hospital, and the validation cohort consisted of patients from Renji Hospital. The distribution of STB was similar, as shown in Figure 1b. Meanwhile, no statistically significant difference (*p* > 0.05) was found in the STB levels among the three centers (Figure 1c). The independent discovery cohort was retrospectively recruited from Huashan Hospital (n = 74). The independent discovery cohort was prospectively recruited from the Shanghai Cancer Center (n = 61). The independent validation cohort was retrospectively recruited from Renji Hospital (n = 94). Table 1 shows the baseline clinical characteristics of the study and validation cohorts. Pearson’s correlation analysis suggested that STB was not significantly correlated with age (r = 0.079, *p* = 0.321) (Supplementary Figure S1).

In the discovery cohort, the mean age was 55.0 years (IQR, 24.0–82.0), and the mean follow-up duration was 20.0 months (IQR, 1.0–60.0). At the last follow-up, 30 (33.7%) patients died, and 56 (62.9%) had confirmed tumor recurrence.

In the validation cohort, the mean age was 59.0 years (IQR, 25.0–88.0), and the mean follow-up duration was 17.0 months (IQR, 1.0–60.0). At the last follow-up, 34 (49.3%) patients died, and 42 (60.9%) had tumor recurrence. The characteristics of the study and validation cohorts were similar with no statistical significance (*p* > 0.05) (Table 2).

### 3.2. Determination of Pretreatment STB and Prognosis of Patients with PCNSL

**Figure 2:**
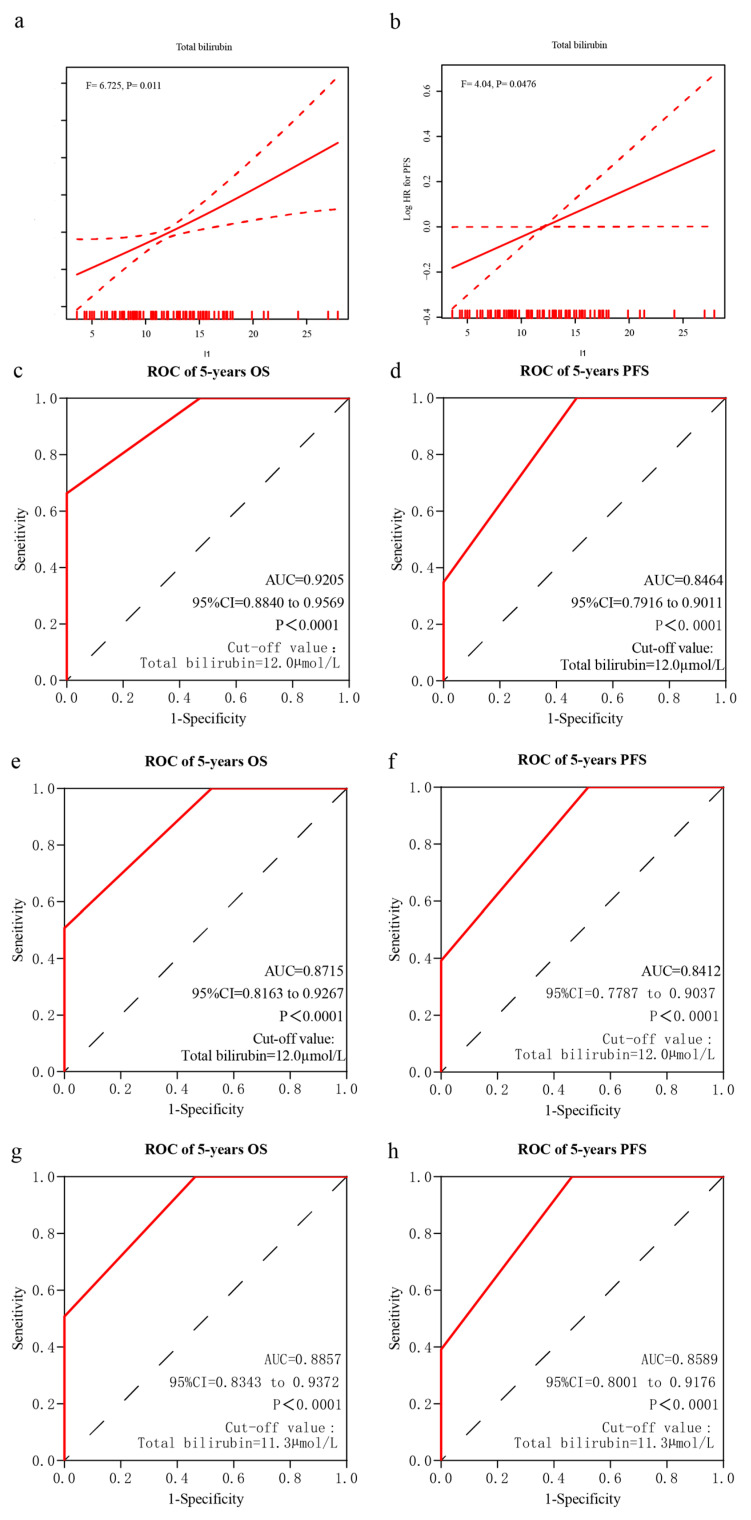
Determination of pretreatment serum total bilirubin (STB) and prognosis of patients with primary central nervous system diffuse large B-cell lymphoma. Generalized additive model plot of the relationship between overall survival (OS) and STB (**a**) and between progression-free survival (PFS) and STB (**b**). Predictive ability of STB in the discovery and validation cohorts. Area under the receiver operating characteristic curve (AUROC) for OS (**c**) and PFS (**d**) in the discovery cohort. AUROC for OS (**e**) and PFS (**f**) in the validation cohort using an STB level of 12.0 µmol/L. AUROC for OS (**g**) and PFS (**h**) in the validation cohort using an STB level of 11.3 µmol/L.

In the discovery cohort, a GAM with Loess was used to identify whether the pretreatment STB levels were associated with the prognosis. The GAM plots revealed a linear relationship between the pretreatment STB levels and clinical outcomes, including OS (Figure 2a, *p* = 0.011) and PFS (Figure 2b, *p* = 0.0476).

Furthermore, the Spearman’s correlation analysis showed that STB was negatively correlated with both OS (r = −0.177, *p* = 0.026) (Supplementary Table S1) and PFS (r = −0.221, *p*= 0.006) (Supplementary Table S2). The logistic regression analysis also suggested that patients with higher STB were more likely to have worse OS (Odds ratio, 1.111; 95% CI, 1.034, 1.193; *p* = 0.004) (Supplementary Table S3).

### 3.3. Comparison of Baseline Characteristics between Patients with STB Levels of <12.0 and ≥12.0 μmol/L in the Discovery Cohort

Patients in the discovery cohort were separated into two groups according to the median STB level of 12.0 µmol/L. The patient characteristics between STB levels of <12.0 and ≥12.0 μmol/L groups in the discovery cohort are presented in Table 3. Compared to patients with STB levels of <12.0 µmol/L, those with STB levels of ≥12.0 μmol/L were more likely to have a higher BMI (*p* = 0.0243) and poorer clinical outcomes (OS, *p* = 0.002; PFS, *p* = 0.132).

### 3.4. Median STB Level as the Cutoff Value

In the discovery cohort, the cutoff value of the STB level for discriminating OS was 12.0 µmol/L with an AUROC of 0.9205 (95% CI, 0.8840–0.9569; *p* < 0.0001) (Figure 2c). In addition, an STB level of 12.0 µmol/L could discriminate PFS with an AUROC of 0.8464 (95% CI, 0.7916–0.9011; *p* < 0.0001) (Figure 2d).

In the validation cohort, the cutoff value of the STB levels for discriminating OS was 11.3 µmol/L. First, we used an STB level of 12.0 µmol/L to discriminate OS and PFS with AUROC values of 0.8715 (95% CI, 0.8163–0.9167; *p* < 0.0001) (Figure 2e) and 0.8412 (95% CI, 0.7787–0.9037; *p* < 0.0001) (Figure 2f), respectively. Second, we used an STB level of 11.3 µmol/L to discriminate OS and PFS with AUROC values of 0.8857 (95% CI, 0.8343–0.9342; *p* < 0.0001) (Figure 2g) and 0.8589 (95% CI, 0.8001–0.9176; *p* < 0.0001) (Figure 2h), respectively.

### 3.5. Prognostic Significance of STB in the Discovery Cohort

**Figure 3:**
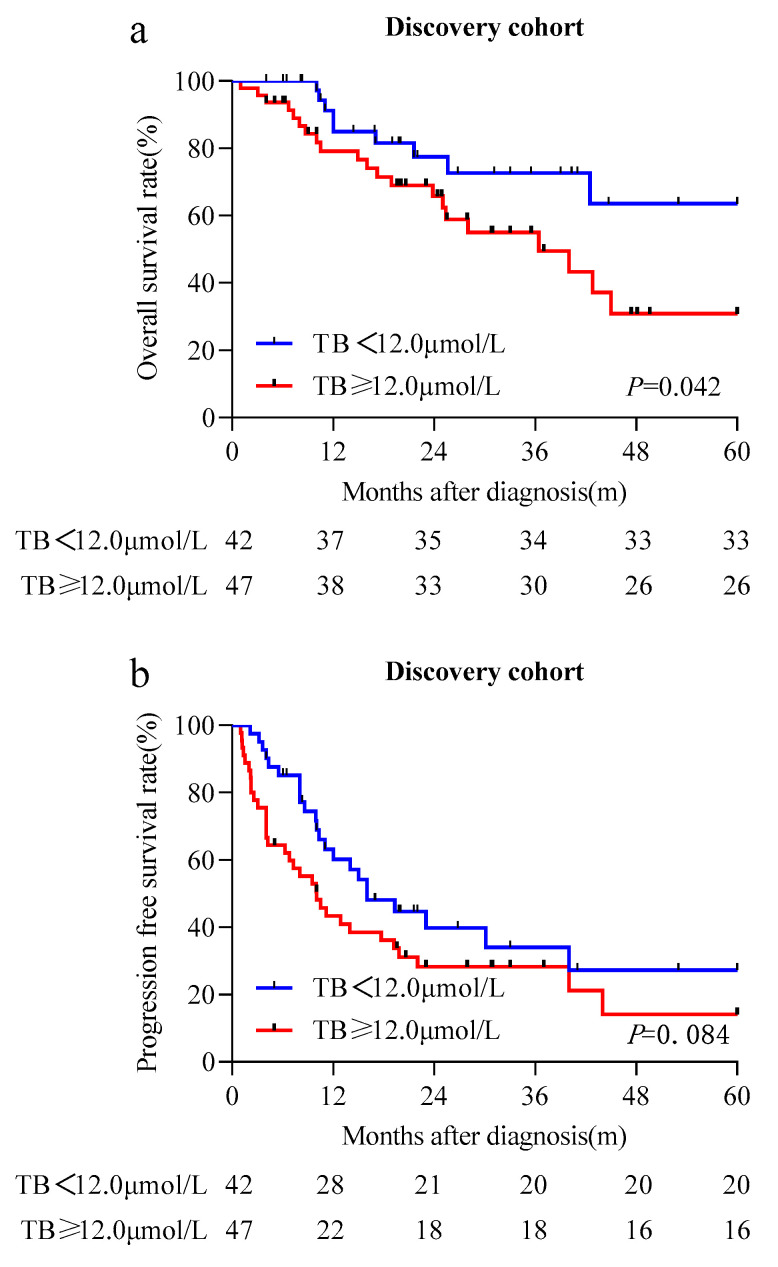
The prognostic significance of the serum total bilirubin (STB) in patients with primary central nervous system diffuse large B-cell lymphoma. Kaplan–Meier curves for overall survival (**a**) and progression-free survival (**b**) in patients who received methotrexate-based chemotherapy categorized by STB in the discovery cohort.

Univariate analysis indicated that age and STB levels were prognostic factors for OS in the discovery cohort (Table 4), whereas sex, diabetes mellitus, hypertension, BMI, and the ALT, AST, and gamma-GT levels had no prognostic significance for OS. Patients with STB levels higher than the median level had significantly lower OS rates (HR, 2.458; 95% CI, 1.087–5.555; *p* = 0.031). Further multivariate analysis confirmed that STB was an independent prognostic factor for OS (HR, 3.912; 95% CI, 1.332–11.493; *p* = 0.013; Table 4). The Kaplan–Meier analysis indicated that the group with STB levels greater than or equal to the median level was associated with significantly lower OS rates (*p* = 0.042) (Figure 3a) and potentially lower PFS rates (*p* = 0.084) (Figure 3b).

Fine and Grey’s competing risk regression model analysis showed that when age was controlled, STB remained independently associated with OS (Sub-distribution HR, 1.0871; 95% CI, 1.0871–1.0871; *p <* 0.0001) (Supplementary Table S4).

### 3.6. Validation of the Prognostic Value of STB in an Independent Cohort

**Figure 4:**
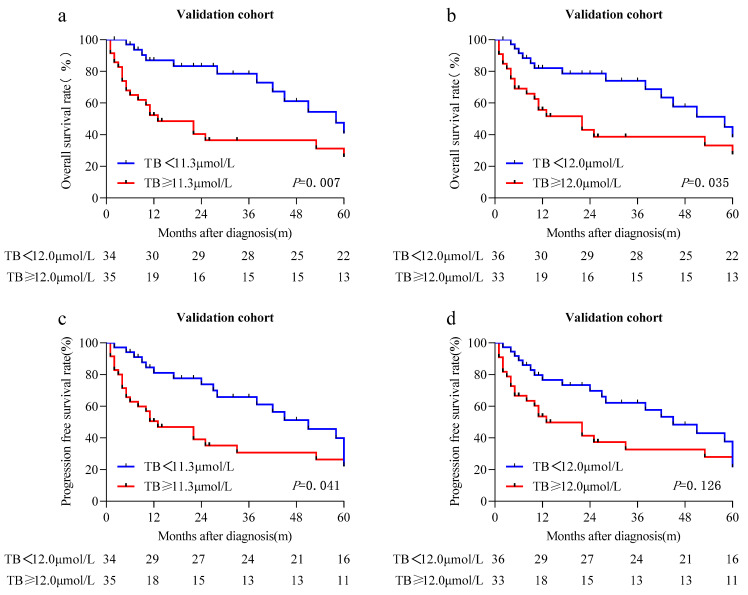
The prognostic significance of serum total bilirubin (STB) in patients with primary central nervous system diffuse large B-cell lymphoma. Kaplan–Meier curve for overall survival and progression-free survival in patients who received methotrexate-based chemotherapy categorized by STB in the validation cohort using the cutoff values of 11.3 µmol/L (**a**,**c**) and 12.0 µmol/L (**b**,**d**).

We further confirmed the prognostic value of the STB levels in an independent validation cohort (n = 69) using the cutoff values of 11.3 and 12.0 µmol/L, respectively, to stratify patients into the high and low STB groups. The results were similar to those found in the discovery cohort, as STB levels greater than or equal to the median levels were also associated with significantly lower OS rates (*p* = 0.007 and *p* = 0.035, respectively) (Figure 4a,b) and PFS rates (when using a cutoff of 11.3 µmol/L, *p* = 0.007) (Figure 4c,d). Univariate analysis indicated that the STB level was significantly associated with OS (HR, 2.525; 95% CI, 1.245–5.121; *p* = 0.010, and HR, 2.043; 95% CI, 1.029–4.055; *p* = 0.041, respectively) (Table 5 and Table 6) and PFS (when using a cutoff of 11.3 µmol/L, HR, 1.855; 95% CI, 1.003–3.432; *p* = 0.049) (Table 6). Further multivariate analysis confirmed that STB was an independent prognostic factor for both OS (HR, 3.671; 95% CI, 1.255–10.734; *p* = 0.018, and HR, 3.061; 95% CI, 1.086–8.625; *p* = 0.034, respectively) (Table 5 and Table 6) and PFS (when using a cutoff of 11.3 µmol/L, HR, 3.174; 95% CI, 1.226–8.215; *p* = 0.017) (Table 6).

## 4. Discussion

Various studies have suggested that serum bilirubin is associated with the occurrence and development of tumors [30,31,32,33,34]. To the best of our knowledge, this is the first study on the relationship between pretreatment STB and the prognosis of patients newly diagnosed with PCNSL who received HD-MTX-based combination immunochemotherapy. In this multicenter prospective–retrospective cohort study, we found that the pretreatment STB was a significant prognostic factor for the OS of patients newly diagnosed with PCNSL who received HD-MTX-based combination immunochemotherapy.

So far, there has not been a consensus on whether STB has a favorable prognostic effect or an unfavorable prognostic effect. Some studies have shown that patients with elevated bilirubin levels tend to have better clinical outcomes or lower cancer risks. For example, Zhang et al. found that in patients with non-small cell lung cancer with EGFR mutations, pretreatment of circulating bilirubin was a significant prognostic factor for OS [30]. Gao et al. reported a positive association between the direct bilirubin level and the risk of lymph node metastasis and a poor prognosis in rectal cancers [31]. Jia et al. also showed that preoperative total bilirubin and direct bilirubin were both identified as potentially significant prognostic factors for the OS of patients with CRC [32]. These findings are consistent with those of the current study.

However, other researchers have reported an inverse correlation between serum bilirubin and cancer survival. In a retrospective study, Cao et al. found that pretreatment STB and direct bilirubin were independent prognostic factors in patients with CRC [33]. In a large-scale prospective study based on data from the UK Biobank, higher levels of pre-diagnostic circulating total bilirubin were significantly inversely associated with the risk of esophageal adenocarcinoma [34]. In a two-sample Mendelian randomization study, researchers found an inverse association between genetically raised bilirubin levels and the risk of squamous cell lung cancer and HL [35]. Possible reasons that may account for the conflicting findings include the following: different sources of patients, different cancer types (solid tumors vs. hematologic tumors), and different statistical methodologies.

The mechanism by which STB may affect patients with PCNSL remains unclear. One possible reason may be UGT1A1 polymorphisms. Glucuronidation mediated by UGT1A1 is an essential step in bilirubin metabolism, and UGT1A1 polymorphisms result in elevated serum UCB levels, known as hyperbilirubinemia. Among them, common and mild unconjugated hyperbilirubinemia is referred to as Gilbert’s syndrome [36], whereas relatively rare and severe hyperbilirubinemia is known as Crigler–Najjar syndrome [37,38]. By potentially altering the ability to metabolize and excrete carcinogens and chemotherapeutic drugs, UGT1A1 polymorphisms can influence both cancer risk and clinical outcomes in different cancer types [39,40]. In particular, a retrospective study reported that in patients with HL, those with the UGT1A1 TA tandem repeat TA6/6 genotype had lower OS (relative risk, 3.63; *p* = 0.004). Currently, there are no other studies investigating UGT1A1 polymorphisms in either lymphoma cancer risk or clinical outcome, let alone PCNSL. Further studies are required to confirm this hypothesis. Another reason may be that higher pretreatment STB levels indicate potential hepatic insufficiency and, thus, patients with these levels are more likely to experience hepatotoxicity caused by HD-MTX. Bilirubin is an important biomarker that reflects hepatic function [41]. Although HD-MTX is mainly eliminated through the kidneys, up to 10% of MTX clearance occurs through hepatic metabolism [42]. Studies have reported an association between HD-MTX therapy and acute/chronic hepatotoxicity, leading to reversible hepatocellular injury [43], and there was a tendency toward higher rates of increase in STB levels in older patients [44]. Although hepatotoxicity caused by HD-MTX alone is not fatal [44], patients with possible abnormal liver functionality can be more sensitive to HD-MTX-related toxicity, especially older patients, resulting in poorer clinical outcomes.

To the best of our knowledge, this is the first prospective study to demonstrate the prognostic value of the pretreatment STB level in PCNSL. Nevertheless, there are several limitations to this study. First, the small number of cases included due to the low incidence of PCNSL may reduce the statistical power of our study. Second, there exist differences in the data collection and methods between the discovery and validation cohorts, making it impossible to analyze certain clinical parameters that may potentially affect outcomes in all patients. Third, although all patients received HD-MTX-based combination immunochemotherapy (HD-MTX+ rituximab), differences between the salvage treatments may also have altered the results in favor of one cohort over the other. Fourth, compared to patients with STB levels lower than the median level, those with STB levels higher than the median level did not have a significantly lower PFS. However, an evident trend was observed. Fifth, while STB was a prognostic factor for clinical outcomes, age might be a confounding factor. However, in our study, multivariate analysis confirmed that STB was still a prognostic after age was corrected. Pearson’s correlation analysis indicated that STB was not significantly correlated with age. Further, Fine and Grey’s competing risk regression model suggested that both STB and age were significantly associated with the prognosis of patients. Together, multivariate analysis, Pearson’s correlation analysis, and Fine and Grey’s competing risk regression model suggested that STB remained an independent prognostic factor after age was controlled. Nevertheless, the possible influence of age on STB cannot be excluded since there exist other potential confounding factors, and it is worthy of further investigation in the future. Lastly, in addition to pathological reasons, the STB level fluctuates due to physiological factors, such as an irregular diet, alcohol consumption, overwork, and staying up late. Meanwhile, laboratory detection methods differ across institutions. Hence, the STB cutoff utilized here may not reflect the exact cutoff, and the reestablishment of the best cutoff value of STB when translating to other institutions is required.

## 5. Conclusions

In summary, this pooled analysis demonstrated that pretreatment STB can be a promising prognostic indicator in patients with PCNSL receiving HD-MTX-based combination immunochemotherapy. This indicates that patients with PCNSL who have an STB level higher than the median level could represent a high-risk population.

## Figures and Tables

**Table 1:** Baseline characteristics of patients in three constitutions.

Variables	All		Huashan Cohort		Shanghai Cancer Center Cohort		Renji Cohort	
	N = 158	%	N = 44%		N = 45%		N = 69	%
**Median age** **(IQR), y**	57 (49, 65)		49 (44.5, 57)		58 (52, 71)		59 (52, 66)	
<60	98	62.0	37	84.0	23	51.1	38	55.1
≥60	60	38.0	7	16.0	22	48.9	31	44.9
**Sex**								
Male	98	62.0	31	70.0	26	57.8	41	59.4
Female	60	38.0	13	30.0	19	42.2	28	40.6
**Diabetes**								
Yes	8	5.1	1	2.3	5	11.1	2	2.9
None	150	94.9	43	97.7	40	88.9	67	97.1
**Hypertension**								
Yes	44	27.8	6	13.6	14	31.1	24	34.8
No	114	72.2	38	86.4	31	68.9	45	65.2
**BMI (IQR),** **kg/m^2^**	23.8 (21.5, 25.8)						23.9 (22.4, 25.4)	
Missing	19	12.0	0	0	2	4.4	17	24.6
<18.0	4	2.5	2	4.6	1	2.2	1	1.4
18.0–24.0	67	42.4	18	40.9	24	53.3	25	36.2
>24.0	68	43.0	24	54.5	18	40.0	26	37.7
**Median Total bilirubin ** **(IQR), µmol/L**	11.9 (8.8, 15.1)		12.0 (8.9, 15.2)		10.6 (8.6, 14.4)		11.3 (9.2, 15.2)	
<12.0	78	49.4	17	28.6	25	55.6	36	52.2
≥12.0	80	50.6	27	61.4	20	44.4	33	47.8
**ALT** **(IQR), U/L**	23.0 (16.0, 28.0)		20.0 (15.0, 25.0)		16.0 (12.0, 25.5)		26.0 (22.0, 33.0)	
**AST** **(IQR), U/L**	21.0 (17.0, 31.0)		21.0 (20.0, 25.0)		18.5 (16.0, 30.0)		26.0 (14.0, 36.0)	
**Gamma-GT** **(IQR), U/L**	21.0 (15.0, 33.0)		21.0 (15.0, 37.0)		18.5 (15.0, 25.5)		23.0 (16.0, 34.0)	

IQR, interquartile range; BMI, body mass index.

**Table 2:** Comparison of the baseline characteristics of the discovery and validation cohorts.

Variables	All		Discovery Cohort		Validation Cohort	
	N = 158	%	N = 89	%	N = 69	%
**Median Age (IQR), y**	57 (49, 65)		55 (48, 62)		59 (52, 66)	
<60	98	62.0	60	67.4	38	55.1
≥60	60	38.0	29	32.6	31	44.9
**Sex**						
Male	98	62.0	57	64.0	41	59.4
Female	60	38.0	32	36.0	28	40.6
**Diabetes**						
Yes	8	5.1	6	6.7	2	2.9
None	150	94.9	83	93.3	67	97.1
**Hypertension**						
Yes	44	27.8	20	22.5	24	34.8
No	114	72.2	69	77.5	45	65.2
**BMI(IQR), kg/m^2^**	23.8 (21.5, 25.8)		23.5 (21.6, 26.1)		23.9 (22.4, 25.4)	
Missing	19	12.0	2	2.2	17	24.6
<18.0	4	2.5	3	3.4	1	1.4
18.0–24.0	67	42.4	42	47.2	25	36.2
>24.0	68	43.0	42	47.2	26	37.7
**Median Total bilirubin ** **(IQR), µmol/L**	11.9 (8.8, 15.1)		12.0 (8.8, 14.5)		11.3 (9.2, 15.2)	
<12.0	78	49.4	42	28.6	47.2	52.2
≥12.0	80	50.6	47	61.4	52.8	47.8
**ALT** **(IQR), U/L**	23.0 (16.0, 28.0)		18.0 (14.0, 25.0)		26.0 (22.0, 33.0)	
**AST** **(IQR), U/L**	21.0 (17.0, 31.0)		20.0 (17.0, 26.0)		26.0 (14.0, 36.0)	
**Gamma-GT** **(IQR), U/L**	21.0 (15.0, 33.0)		20.0 (15.0, 30.0)		23.0 (16.0, 34.0)	

**Table 3:** Comparison of the baseline characteristics between patients with serum total bilirubin levels of <12.0 and ≥12 μmol/L in the discovery cohort.

Variables	All		Total Bilirubin < 12.0		Total Bilirubin ≥ 12.0		
	N = 89	%	N = 44	%	N = 45	%	*p*
**Discovery cohort** **Median age (IQR), y**	55 (48, 62)		49 (44.5, 57)		58 (52, 71)		
<60	60	67.4	37	84.0	23	51.1	0.887
≥60	29	32.6	7	16.0	22	48.9	
**Sex**							
Male	57	64.0	31	70.0	26	57.8	0.009
Female	32	36.0	13	30.0	19	42.2	
**Diabetes**							
Yes	6	6.7	1	2.3	5	11.1	0.571
None	83	93.3	43	97.7	40	88.9	
**Hypertension**							
Yes	20	22.5	6	13.6	14	31.1	0.215
No	69	77.5	38	86.4	31	68.9	
**BMI, kg/ m^2^**	23.5 (21.6, 26.1)						
Missing	2	2.2	0	0	2	4.4	0.0243
<18.0	3	3.4	2	4.6	1	2.2	
18.0–24.0	42	47.2	18	40.9	24	53.3	
>24.0	42	47.2	24	54.5	18	40.0	
**Death**							
Yes	30	33.7	9	21.4	21	44.7	0.002
None	59	66.3	33	78.6	26	55.3	
**Progression**							
Yes	56	62.9	23	54.8	33	70.2	0.132
None	30	37.1	19	45.2	14	29.8	
**ALT** **(IQR), U/L**	18.0 (14.0, 25.0)		16.0 (11.5, 27.0)		19.0 (15.0, 24.0)		0.241
**AST** **(IQR), U/L**	20.0 (17.0, 26.0)		19.0 (15.5, 31.0)		20.5 (20.0, 25.0)		0.182
**Gamma-GT** **(IQR), U/L**	20.0 (15.0, 30.0)		20.0 (14.5, 28.0)		20.0 (15.0, 34.0)		0.511

**Table 4:** Univariate and multivariate analyses of overall survival (OS) and progression-free survival (PFS) in the discovery cohort.

	OS	PFS		
	HR (95% CI)	*p*	HR (95% CI)	*p*
**Univariate analysis**				
**Age, y**	1.061 (1.024, 1.100)	0.001	1.015 (0.991, 1.039)	0.221
**Sex (male vs. female)**	1.053 (0.487, 2.276)	0.895	1.158 (0.663, 2.022)	0.606
**Diabetes (yes vs. no)**	2.148 (0.492, 9.378)	0.309	1.849 (0.731, 4.680)	0.194
**Hypertension (yes vs. no)**	1.215 (0.515, 2.865)	0.657	1.089 (0.580, 2.046)	0.790
**BMI, kg/ m^2^**	1.011 (0.901, 1.133)	0.858	1.050 (0.969, 1.138)	0.230
**ALT, U/L**	1.022 (0.993, 1.053)	0.138	1.009 (0.989, 1.029)	0.391
**AST, U/L**	1.026 (0.992, 1.062)	0.138	1.011 (0.988, 1.036)	0.351
**Gamma-GT, U/L**	0.148 (0.995, 1.030)	1.030	1.005 (0.993, 1.018)	0.412
**Total bilirubin, µmol/L** **(≥12.0 vs. <12.0)**	2.458 (1.087, 5.555)	0.031	1.637 (0.954, 2.810)	0.074
**Multivariate analysis**				
**Age, y**	1.082 (1.037, 1.128)	<0.001	1.015 (0.989, 1.042)	0.266
**Sex (male vs. female)**	0.978 (0.405, 2.361)	0.960	1.193 (0.642, 2.217)	0.577
**Diabetes (yes vs. no)**	1.505 (0.271, 8.371)	0.640	1.701 (0.577, 5.020)	0.336
**Hypertension (yes vs. no)**	0.804 (0.311, 2.078)	0.652	0.991 (0.487, 2.014)	0.980
**BMI, kg/ m^2^**	1.059 (0.937, 1.196)	0.362	1.053 (0.965, 1.149)	0.249
**ALT, U/L**	0.961 (0.891, 1.036)	0.295	0.992 (0.942, 1.044)	0.744
**AST, U/L**	1.029 (0.943, 1.124)	0.516	1.017 (0.960, 1.077)	0.567
**Gamma-GT, U/L**	1.021 (0.995, 1.047)	0.119	1.002 (0.986, 1.019)	0.796
**Total bilirubin, µmol/L** **(≥12.0 vs. <12.0)**	3.912 (1.332, 11.493)	0.013	1.957 (1.042, 3.675)	0.037

**Table 5:** Univariate and multivariate analyses of overall survival (OS) and progression-free survival (PFS) in the validation cohort using the cutoff value of 12.0 µmol/L.

Variables	OS		PFS	
	HR (95% CI)	*p*	HR (95% CI)	*p*
**Univariate analysis**				
**Age, y**	1.039 (1.004, 1.074)	0.026	1.028 (0.998, 1.059)	0.064
**Sex (male vs. female)**	0.959 (0.483, 1.902)	0.904	0.594 (0.322, 1.096)	0.095
**Diabetes (yes vs. no)**	0.698 (0.095, 5.125)	0.724	0.555 (0.076, 4.046)	0.561
**Hypertension (yes vs. no)**	1.260 (0.636, 2.496)	0.508	1.114 (0.600,2.070)	0.732
**BMI, kg/m^2^**	1.073 (0.954, 1.207)	0.240	1.065 (0.956, 1.188)	0.253
**ALT, U/L**	0.991 (0.950, 1.034)	0.684	1.011 (0.975, 1.049)	0.545
**AST, U/L**	0.991 (0.975, 1.007)	0.275	0.997 (0.986, 1.008)	0.602
**Gamma-GT, U/L**	0.999 (0.977, 1.022)	0.933	1.006 (0.987, 1.025)	0.537
**Total bilirubin, µmol/L** **(≥12.0 vs. <12.0)**	1.039 (1.004, 1.074)	0.041	1.028 (0.998, 1.059)	0.137
**Multivariate analysis**				
**Age, y**	1.030 (0.980, 1.082)	0.249	1.019 (0.974, 1.066)	0.414
**Sex (male vs. female)**	1.302 (0.389, 4.354)	0.669	0.650 (0.225, 1.875)	0.425
**Diabetes (yes vs. no)**	0.273 (0.016, 4.754)	0.374	0.515 (0.037, 7.178)	0.621
**Hypertension (yes vs. no)**	1.704 (0.559, 5.198)	0.349	1.214 (0.425, 3.464)	0.718
**BMI, kg/m^2^**	1.142 (0.937, 1.392)	0.189	1.102 (0.921, 1.320)	0.289
**ALT, U/L**	1.060 (0.958, 1.173)	0.258	1.053 (0.968, 1.147)	0.230
**AST, U/L**	1.002 (0.957, 1.049)	0.934	0.995 (0.958, 1.033)	0.786
**Gamma-GT, U/L**	0.984 (0.925, 1.047)	0.607	1.002 (0.950, 1.057)	0.938
**Total bilirubin, µmol/L** **(≥12.0 vs. <12.0)**	3.671 (1.255, 10.734)	0.018	2.627 (1.048, 6.584)	0.039

**Table 6:** Univariate and multivariate analyses of overall survival (OS) and progression-free survival (PFS) in the validation cohort using the cutoff value of 11.3 µmol/L.

Variables	OS		PFS	
	HR (95% CI)	*p*	HR (95% CI)	*p*
**Univariate analysis**				
**Age, y**	1.039 (1.004, 1.074)	0.026	1.028 (0.998, 1.059)	0.064
**Sex (male vs. female)**	0.959 (0.483, 1.902)	0.904	0.594 (0.322, 1.096)	0.095
**Diabetes (yes vs. no)**	0.698 (0.095, 5.125)	0.724	0.555 (0.076, 4.046)	0.561
**Hypertension (yes vs. no)**	1.260 (0.636, 2.496)	0.508	1. 114 (0.600, 2.070)	0.732
**BMI, kg/m^2^**	1.073 (0.954, 1.207)	0.240	1.065 (0.956, 1.188)	0.253
**ALT, U/L**	0.991 (0.950, 1.034)	0.684	1.011 (0.975, 1.049)	0.545
**AST, U/L**	0.991 (0.975, 1.007)	0.275	0.997 (0.986, 1.008)	0.602
**Gamma-GT, U/L**	0.999 (0.977, 1.022)	0.933	1.006 (0.987, 1.025)	0.537
**Total bilirubin, µmol/L** **(≥ 11.3 vs. <11.3)**	2.525 (1.245, 5.121)	0.010	1.855 (1.003, 3.432)	0.049
**Multivariate analysis**				
**Age, y**	1.024 (0.975, 1.076)	0.335	1.022 (0.976, 1.070)	0.364
**Sex (male vs. female)**	1.493 (0.425, 5.251)	0.532	0.596 (0.209, 1.703)	0.334
**Diabetes (yes vs. no)**	0.257 (0.014, 4.662)	0.257	0.518 (0.037, 7.239)	0.625
**Hypertension (yes vs. no)**	2.039 (0.642, 6.478)	0.227	1.084 (0.386, 3.045)	0.878
**BMI, kg/ m^2^**	1.168 (0.954, 1.430)	0.134	1.085 (0.905, 1.299)	0.378
**ALT, U/L**	1.068 (0.963, 1.183)	0.211	1.052 (0.966, 1.145)	0.246
**AST, U/L**	1.005 (0.960, 1.052)	0.834	0.994 (0.956, 1.033)	0.753
**Gamma-GT, U/L**	0.978 (0.919, 1.041)	0.482	1.005 (0.953, 1.060)	0.844
**Total bilirubin, µmol/L** **(≥11.3 vs. <11.3)**	3.061 (1.086, 8.625)	0.034	3.174 (1.226, 8.215)	0.017

## Data Availability

The datasets used and/or analyzed during the current study are available from the corresponding author on reasonable request.

## Supplementary Materials

The following supporting information can be downloaded at: https://www.mdpi.com/article/10.3390/cancers15184584/s1, Table S1: Spearman correlation analysis on association between serum total bilirubin and overall survival (OS). Table S2: Spearman correlation analysis on association between serum total bilirubin and progression-free survival (PFS). Table S3: Logistic regression result on association between serum total bilirubin and clinical outcomes. Table S4: Fine and Grey competing risk regression model analysis for overall survival (OS) in discovery cohort. Figure S1: pearson correlation analysis on relationship between serum total bilirubin and age.

## References

[1] Löw S., Han C.H., Batchelor T.T. (2018). Primary central nervous system lymphoma. Ther. Adv. Neurol. Disord..

[2] Yuan Y., Ding T., Wang S., Chen H., Mao Y., Chen T. (2021). Current and emerging therapies for primary central nervous system lymphoma. Biomark. Res..

[3] Langner-Lemercier S., Houillier C., Soussain C., Ghesquières H., Chinot O., Taillandier L., Soubeyran P., Lamy T., Morschhauser F., Benouaich-Amiel A. (2016). Primary CNS lymphoma at first relapse/progression: Characteristics, management, and outcome of 256 patients from the French LOC network. Neuro Oncol..

[4] Calimeri T., Steffanoni S., Gagliardi F., Chiara A., Ferreri A.J.M. (2021). How we treat primary central nervous system lymphoma. ESMO Open.

[5] Schaff L.R., Grommes C. (2022). Primary Central Nervous System Lymphoma. Blood.

[6] Ferreri A.J., Blay J.Y., Reni M., Pasini F., Spina M., Ambrosetti A., Calderoni A., Rossi A., Vavassori V., Conconi A. (2003). Prognostic scoring system for primary CNS lymphomas: The International Extranoda Lymphoma Study Group experience. J. Clin. Oncol..

[7] Abrey L.E., Ben-Porat L., Panageas K.S., Yahalom J., Berkey B., Curran W., Schultz C., Leibel S., Nelson D., Mehta M. (2006). Primary central nervous system lymphoma: The Memorial Sloan-Kettering Cancer Center prognostic model. J. Clin. Oncol..

[8] Fevery J. (2008). Bilirubin in clinical practice: A review. Liver Int..

[9] Sticova E., Jirsa M. (2013). New insights in bilirubin metabolism and their clinical implications. World J. Gastroenterol..

[10] Čvorovic J., Passamonti S. (2017). Membrane Transporters for Bilirubin and Its Conjugates: A Systematic Review. Front. Pharmacol..

[11] Hofmann A.F. (1999). Bile acids, cholesterol, gallstone calcification, and the enterohepatic circulation of bilirubin. Gastroenterology.

[12] Stocker R., Yamamoto Y., McDonagh A.F., Glazer A.N., Ames B.N. (1987). Bilirubin is an antioxidant of possible physiological importance. Science.

[13] Najib F. (1937). Defensive Role of BilirubinÆ Mia in Pneumococcal Infection. Lancet.

[14] Wallner M., Marculescu R., Doberer D., Wolzt M., Wagner O., Vitek L., Bulmer A.C., Wagner K.H. (2013). Protection from age-related increase in lipid biomarkers and inflammation contributes to cardiovascular protection in Gilbert’s syndrome. Clin. Sci..

[15] Hou L., Li H., Si S., Yu Y., Sun X., Liu X., Yan R., Yu Y., Wang C., Yang F. (2021). Exploring the causal pathway from bilirubin to CVD and diabetes in the UK biobank cohort study: Observational findings and Mendelian randomization studies. Atherosclerosis.

[16] Kwon Y.J., Lee Y.J., Park B.J., Hong K.W., Jung D.H. (2018). Total serum bilirubin and 8-year incident type 2 diabetes mellitus: The Korean Genome and Epidemiology Study. Diabetes Metab..

[17] Bianco A., Tiribelli C., Bellarosa C. (2022). Translational Approach to the Protective Effect of Bilirubin in Diabetic Kidney Disease. Biomedicines.

[18] Wagner K.H., Wallner M., Mölzer C., Gazzin S., Bulmer A.C., Tiribelli C., Vitek L. (2015). Looking to the horizon: The role of bilirubin in the development and prevention of age-related chronic diseases. Clin. Sci..

[19] Adin C.A. (2021). Bilirubin as a Therapeutic Molecule: Challenges and Opportunities. Antioxidants.

[20] Mantovani A., Allavena P., Sica A., Balkwill F. (2008). Cancer-related inflammation. Nature.

[21] Seyed Khoei N., Jenab M., Murphy N., Banbury B.L., Carreras-Torres R., Viallon V., Kühn T., Bueno-de-Mesquita B., Aleksandrova K., Cross A.J. (2020). Circulating bilirubin levels and risk of colorectal cancer: Serological and Mendelian randomization analyses. BMC Med..

[22] Ioannou G.N., Liou I.W., Weiss N.S. (2006). Serum bilirubin and colorectal cancer risk: A population-based cohort study. Aliment. Pharmacol. Ther..

[23] Monroy-Iglesias M.J., Moss C., Beckmann K., Hammar N., Walldius G., Bosco C., Van Hemelrijck M., Santaolalla A. (2021). Serum Total Bilirubin and Risk of Cancer: A Swedish Cohort Study and Meta-Analysis. Cancers.

[24] Li D., Li S., Xia Z., Cao J., Zhang J., Chen B., Zhang X., Zhu W., Fang J., Liu Q. (2022). Prognostic significance of pretreatment red blood cell distribution width in primary diffuse large B-cell lymphoma of the central nervous system for 3P medical approaches in multiple cohorts. EPMA J..

[25] Li S., Xia Z., Cao J., Zhang J., Chen B., Chen T., Zhang X., Zhu W., Li D., Hua W. (2022). Proposed new prognostic model using the systemic immune-inflammation index for primary central nervous system lymphoma: A prospective-retrospective multicohort analysis. Front. Immunol..

[26] Swerdlow S.H., Campo E., Pileri S.A., Harris N.L., Stein H., Siebert R., Advani R., Ghielmini M., Salles G.A., Zelenetz A.D. (2016). The 2016 revision of the World Health Organization classification of lymphoid neoplasms. Blood.

[27] Li Q., Ma J., Ma Y., Lin Z., Kang H., Chen B. (2021). Improvement of outcomes of an escalated high-dose methotrexate-based regimen for patients with newly diagnosed primary central nervous system lymphoma: A real-world cohort study. Cancer Manag. Res..

[28] Hastie T., Tibshirani R. (1995). Generalized additive modelsfor medical research. Stat. Methods Med. Res..

[29] Wood S.N. (2017). Generalized Additive Models: An Introduction with R.

[30] Zhang Y., Xu J., Lou Y., Hu S., Yu K., Li R., Zhang X., Jin B., Han B. (2017). Pretreatment direct bilirubin and total cholesterol are significant predictors of overall survival in advanced non-small-cell lung cancer patients with EGFR mutations. Int. J. Cancer.

[31] Gao C., Fang L., Li J.T., Zhao H.C. (2016). Significance and prognostic value of increased serum direct bilirubin level for lymph node metastasis in Chinese rectal cancer patients. World J. Gastroenterol..

[32] Jia Z., Zhu Z., Wang Y., Ding J., Lin Z., Zhang Y., Li Z. (2021). The prognostic value of serum bilirubin in colorectal cancer patients with surgical resection. Int. J. Biol. Markers.

[33] Cao Y., Deng S., Yan L., Gu J., Yang J., Yang M., Liu L., Cai K. (2021). A nomogram based on pretreatment levels of serum bilirubin and total bile acid levels predicts survival in colorectal cancer patients. BMC Cancer.

[34] Seyed Khoei N., Wagner K.H., Carreras-Torres R., Gunter M.J., Murphy N., Freisling H. (2021). Associations between Prediagnostic Circulating Bilirubin Levels and Risk of Gastrointestinal Cancers in the UK Biobank. Cancers.

[35] Seyed Khoei N., Carreras-Torres R., Murphy N., Gunter M.J., Brennan P., Smith-Byrne K., Mariosa D., Mckay J., O’Mara T.A., Jarrett R. (2021). Genetically Raised Circulating Bilirubin Levels and Risk of Ten Cancers: A Mendelian Randomization Study. Cells.

[36] Wagner K.H., Shiels R.G., Lang C.A., Seyed Khoei N., Bulmer A.C. (2018). Diagnostic criteria and contributors to Gilbert’s syndrome. Crit. Rev. Clin. Lab. Sci..

[37] Maruo Y., Nakahara S., Yanagi T., Nomura A., Mimura Y., Matsui K., Sato H., Takeuchi Y. (2016). Genotype of UGT1A1 and phenotype correlation between Crigler-Najar syndrome type II and Gilbert syndrome. J. Gastroenterol. Hepatol..

[38] Dhawan A., Lawlor M.W., Mazariegos G.V., McKiernan P., Squires J.E., Strauss K.A., Gupta D., James E., Prasad S. (2020). Disease burden of Crigler-Najar syndrome: Systematic review and future perspectives. J. Gastroenterol. Hepatol..

[39] Takano M., Sugiyama T. (2017). UGT1A1 polymorphisms in cancer: Impact on irinotecan treatment. Pharmgenom. Pers. Med..

[40] Nelson R.S., Seligson N.D., Bottiglieri S., Carballido E., Cueto A.D., Imanirad I., Levine R., Parker A.S., Swain S.M., Tillman E.M. (2021). UGT1A1 Guided Cancer Therapy: Review of the Evidence and Considerations for Clinical Implementation. Cancers.

[41] Memon N., Weinberger B.I., Hegyi T., Aleksunes L.M. (2016). Inherited disorders of bilirubin clearance. Pediatr. Res..

[42] DeFino C.E., Barreto J.N., Pawlenty A.G., Ruff M.W., Carabenciov I.D., Mara K.C., Thompson C.A. (2021). Lack of drug interaction between levetiracetam and high-dose methotrexate in patients with lymphoma. Pharmacotherapy.

[43] Green M.R., Chowdhary S., Lombardi K.M., Chalmers L.M., Chamberlain M. (2006). Clinical utility and pharmacology of high-dose methotrexate in the treatment of primary CNS lymphoma. Expert Rev. Neurother..

[44] Jahnke K., Korfel A., Martus P., Weller M., Herrlinger U., Schmittel A., Fischer L., Thiel E., German Primary Central Nervous System Lymphoma Study Group (G-PCNSL-SG) (2005). High-dose methotrexate toxicity in elderly patients with primary central nervous system lymphoma. Ann. Oncol..

