# Supplementary material for: Development and Validation of Pretreatment Serum Total Bilirubin as a Biomarker to Predict the Clinical Outcomes in Primary Central Nervous System Lymphoma: A Multicenter Cohort Study
Source: Cancers (Basel). 2023 Sep 15;15(18):4584. doi: 10.3390/cancers15184584 (PMC10526312; doi:10.3390/cancers15184584)

Supplementary Table S1. Spearman correlation analysis on association between serum total bilirubin and overall survival (OS).

Correlations

			**Total bilirubin**	**OS**
**Spearman's rho**	**Total bilirubin, µmol/L**	correlation coefficient	1.000	-.177*
		Sig.(2-tailed)	.	.026
		N	158	158
	**OS**	correlation coefficient	-.177*	1.000
		Sig.(2-tailed)	.026	.
		N	158	158

*. Correlation is significant at the 0.01 level (2-tailed).

Supplementary Table S2. Spearman correlation analysis on association between serum total bilirubin and progression-free survival (PFS).

Correlations

			**Total bilirubin**	**PFS**
**Spearman's rho**	**Total bilirubin, µmol/L**	correlation coefficient	1.000	-.221**
		Sig.(2-tailed)	.	.006
		N	158	155
	**OS**	correlation coefficient	-.221**	1.000
		Sig.(2-tailed)	.006	.
		N	155	155

**. Correlation is significant at the 0.01 level (2-tailed).

Supplementary Table S3. Logistic regression result on association between serum total bilirubin and clinical outcomes.

**Variables**	**OS**		**PFS**	
	**Odds Ratio (95% CI)**	***P***	**Odds Ratio (95% CI)**	***P***
**Total bilirubin, µmol/L**	1.111 (1.034, 1.193)	0.004	1.070 (0.995, 1.151)	0.068

Supplementary Table S4. Fine and Grey competing risk regression model analysis for overall survival (OS) in discovery cohort.

Constant effects	Sub-distribution HR	95% CI low	95% CI up	*P*	Coefficient	Se	Z
**age**	1.0871	1.0871	1.0871	＜0.0001	0.08355	0.00000	Inf
**Total bilirubin, µmol/L**	1.2101	1.2101	1.2101	＜0.0001	0.19069	0.00000	Inf

Supplementary Figure S1. pearson correlation analysis on relationship between serum total bilirubin and age.

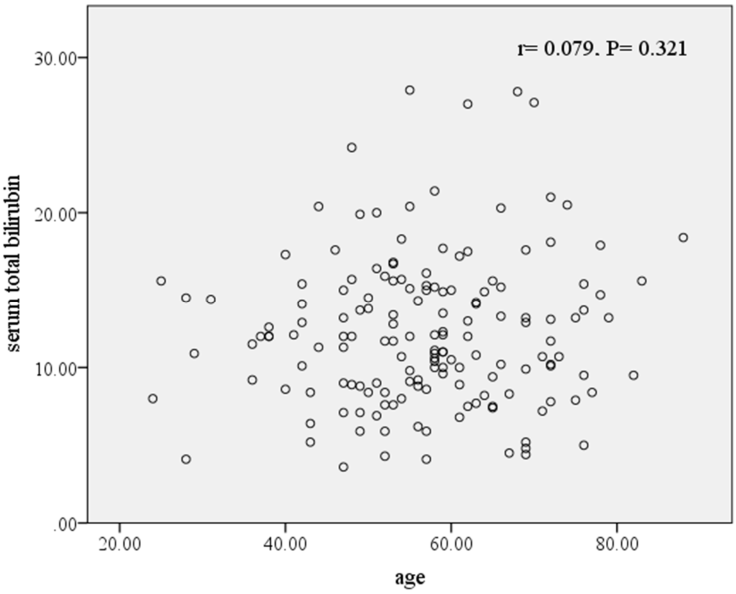

Supplement: Supplementary file 1 [file cancers-15-04584-s001.zip › cancers-2569979-supplementary.pdf]
